# Work for the Wounded of the Allied Armies

**Published:** 1914-12-05

**Authors:** 


### HOSPITALS AND THE WAR.

(From our Special Correspondents.)

### Work for the Wounded of the Allied Armies.

### BRADFORD.

On Thursday, November 26, information was received that a convoy of wounded would reach Leeds, arriving at Wellington Station at 6.30 o'clock that evening, and that forty cases would be sent to Bradford. A fleet of motor-cars, kindly lent for the purpose, met the ambulance train at Leeds, and brought the soldiers to Bradford, where twenty were allocated to the Royal Infirmary and twenty to the Royal Eye and Ear Hospital. The ambulance train had left Southampton at 10.30 a.m., and the men looked somewhat tired after the journey. A bath and a good meal were much appreciated by them.

At Woodlands Convalescent Home, Rawdon, an adjunct of the Bradford Royal Infirmary, there are now twenty-seven British and fifty-eight Belgian soldiers under treatment.

### BRISTOL.

Sixty British and Belgian wounded arrived at Bristol on November 23, of whom forty were sent to Southmead and twenty to the Royal Infirmary; 105 also came on the Thursday last week (26th), including thirty-three "stretcher" cases. Since the outbreak of the war over 2,400 patients have been received at the base hospital.

The local branch of the Red Cross Society has decided to open an additional "reception room" for the allocation of gifts and stores. A portion of the Bristol Cemetery has also been reserved for the interment of those dying in hospital.

### CARDIFF: VICTIMS OF "GENERAL WINTER."

Another batch of 100 soldiers arrived at Cardiff on Sunday last (November 29) from Southampton, making 426 in three weeks. Though dismal weather prevailed and their coming was unexpected there were large crowds to greet them. The men travelled from Southampton by the Welsh R.A.M.C train, and their transportation and conveyance were accomplished with customary expedition under the direction of Major E. Maclean and Commandant J. Sparrow. Twenty-five were "cot" cases; whilst seventy-five were described as "sick." The new hardships with which the overseas troops are now having to contend was revealed by the presence of a large number of men suffering from frostbite; there was also a good percentage of cases of rheumatism. The men were allocated, as to twenty-five, to the Howard Gardens; thirty-seven to the Albany Road, and thirty-eight to the Splott Road sections of the hospital. It was interesting to note the presence of three Territorials, including the second member of the London Scottish Regiment who has been conveyed to Cardiff, and of one of the Royal Flying Corps.

The soldiers manifested their customary cheerfulness, and the remark of one of them as he passed on a stretcher, "Well, it's better coming this way than not at all!" was certainly typical. One man said that on one occasion a sleeve and a portion of his tunic were torn off by shrapnel; whilst on another he was hurled into a hedge. The 3rd Western General Hospital, in short, has been as busy as ever.

### LEICESTER: ARRIVAL OF MEDICAL CASES.

The announcement that a further convoy of sick and wounded English soldiers was being dispatched from Southampton last Sunday was not unwelcome after a period of two weeks' comparative quietude from admissions to the 5th Northern General Hospital. Numerous transfers of patients had recently been effected to the various convalescent homes and Voluntary Aid Detachment centres, and to the Groby Road (Leicester) Isolation Hospital, with the result that the number of patients had been reduced to about half the available bed accommodation. The new convoy left Southampton at 4.35, and the ambulance train arrived with 114 patients at 10.35.

Contrary to experience with earlier convoys, which disclosed few medical cases, these preponderated with the present batch of patients, and very few suffered from wounds. The transport arrangements were under the supervision of Mr. A. W. Faire, County Director, V.A.D. Twenty patients were conveyed to the Leicester Royal Infirmary and the remainder to the base hospital. Many of the men were suffering from the effects of frostbite, bronchitis, and pneumonia.

### NORFOLK AND NORWICH HOSPITAL: THE DIRECTOR OF MEDICAL SERVICES' TRIBUTE.

Already five convoys have arrived, making 500 in all, since war was declared, and to enable the hospital to take in 100 cases per week the board of management have undertaken to put up temporary accommodation for sixty beds in addition to the 165 beds already provided for the sick and wounded soldiers.

To enable this additional undertaking to be carried out the *Eastern Daily Press* has made itself responsible for collecting 2,000 guineas to pay for the cost, and three days after the subscription list opened more than three parts of the amount had been subscribed. The following letter from the Director of Medical Services, Eastern Command, has been received by Mr. F. G. Hazell, the secretary, and it will be seen that it gives the Norfolk and Norwich Hospital full credit for the excellent work it is carrying out:—

Horse Guards, Whitehall, London, S.W.
November 14, 1914.

Sir,—I beg to acknowledge your letter of the 13th inst. I should welcome on behalf of the General Officer Commanding-in-Chief, Eastern Command, any suggestion to enlarge the accommodation of the Norfolk and Norwich Hospital. We shall, I feel sure, require every bed we can get placed at our disposal by such institutions as your hospital for sick and wounded from the Front. A hospital of your character is a very great help to us, as you are in a position in your admirably equipped institution, and with your well-trained and capable staff, to take full control of direct convoys of sick and wounded from the Front.

I was very glad to have had an opportunity personally of seeing your hospital, as I now feel entire confidence that our casualties from the Front will receive the best treatment in your institution. I beg to thank you on behalf of the military authorities for the great assistance you are giving to our sick and wounded, and I am sure you will agree with me that we cannot do too much for our gallant soldiers who have spent themselves so readily in the services of the nation.—I am, Sir, your obedient servant,

(Signed) H. R. Whitehead, Surgeon-General,
Deputy Director of Medical Services,
Eastern Command.

### TUNBRIDGE WELLS: A NEW PROBLEM.

There has been a constant stream of patients coming from Chatham to both the General and to the Eye and Ear Hospitals, the latter hospital doing good service in eye cases, and there has been a constant stream of cured patients going from the hospitals to be ready in quite a short time for the fighting line again.

As the War Office does not recognise convalescent homes for Belgian soldiers and requires the hospitals to keep them until fit for active service, a new problem has arisen which the committee is trying to solve by obtaining the services of a number of gentlemen to act as conductors to the men during exercise.

### BATH: HOSPITAL VISITS AT 1s. A HEAD.

Further contingents of soldiers—both British and Belgian—suffering from rheumatism continue to arrive in Bath, and up to the present time some forty patients have been received at the Royal Mineral Water Hospital, while a number are being accommodated at the temporary hospital at Kingswood School, Lansdown, and are motored down to the city daily for treatment. The Bath motor ambulance for the Front, provided by public subscription, on Friday last week (November 27) was officially handed over to the St. John Ambulance Association. It is a "four-stretcher" body with a 14-20 h.p. chassis. The Bath Women's Voluntary Aid Detachment has mobilised and opened a Red Cross hospital at 9 Lansdown Place. The larger rooms have been converted into the wards, the lockers for which have been made by the boys of the Somerset Industrial School, and there is accommodation for about thirty patients; a bathroom is available on each floor, and that which answers to the usual ward kitchen exists at the top of the building.

At the present moment the hospital is being used for the reception of any soldiers in training at Bath who may be in need of treatment, and a small number of bronchial cases have been received. The commandant is Dr. Lindsay, and the lady superintendent Miss Hill. The hospital is fortunate in possessing its own motor ambulance, but has embarked upon what is certainly a novel and, the writer believes, unheard-of policy of charging the sum of one shilling to all visitors who desire to see over the hospital!

### WALSALL: EQUIPMENT OF MILITARY AND VOLUNTARY HOSPITALS.

The committee of the Walsall and District Hospital, in August last, offered to place at the disposal of the military authorities thirty beds for the accommodation of wounded soldiers, and a few weeks ago an intimation was received from the headquarters of the Northern Command at York that these beds had been placed at the disposal of the Military Hospital, Lichfield. On November 24 five soldiers were transferred from Lichfield to Walsall, in order that they might have the benefit of the *x*-ray department attached to the hospital—the Military Hospital having no apparatus of this description. Skiagrams were taken, and the men returned to Lichfield on Thursday last week.

Everyone in the hospital did their utmost to make the short stay of these men as comfortable as possible, and the statement of your correspondent at Leeds—that the men who have had experience of both military and civilian hospitals are strong in their expression of appreciation of the advantages and freedom of the latter—is endorsed by the statements of these men, who were very desirous of remaining at Walsall for a few more days.

